# Significant Mobility of Novel Heteroaggregates of Montmorillonite Microparticles with Nanoscale Zerovalent Irons in Saturated Porous Media

**DOI:** 10.3390/toxics10060332

**Published:** 2022-06-17

**Authors:** Chongyang Shen, Jinan Teng, Wenjuan Zheng, Dong Liu, Ke Ma

**Affiliations:** 1 Key Laboratory of Degraded and Unused Land Consolidation Engineering, Ministry of Natural Resources of the People’s Republic of China, Xi’an 710075, China; 2 Department of Soil and Water Sciences, China Agricultural University, Beijing 100193, China; njt54326@163.com (J.T.); liudong@cau.edu.cn (D.L.); make_linda@163.com (K.M.); 3 Department of Mechanics and Aerospace Engineering, Southern University of Science and Technology, Shenzhen 518055, China; zhengwj@mail.sustech.edu.cn

**Keywords:** nanoscale zerovalent iron, montmorillonite, transport, porous media, remediation

## Abstract

This study conducted laboratory column experiments to systematically examine the transport of novel heteroaggregates of montmorillonite (Mt) microparticles with nanoscale zerovalent irons (nZVIs) in saturated sand at solution ionic strengths (ISs) ranging from 0.001 to 0.2 M. Spherical nZVIs were synthesized using the liquid phase reduction method and were attached on the plate-shaped Mt surfaces in monolayer. While complete deposition occurred for nZVIs in sand, significant transport was observed for Mt-nZVI heteroaggregates at IS ≤ 0.01 M despite the transport decrease with an increasing loading concentration of nZVIs on Mt. The increased mobility of Mt-nZVI heteroaggregates was because the attractions between nZVIs and sand collectors were reduced by the electrostatic repulsions between the Mt and the collector surfaces, which led to a decreased deposition in the sand columns. Complete deposition occurred for the Mt-nZVI heteroaggregates at IS ≥ 0.1 M due to a favorable deposition at Derjaguin–Landau–Verwey–Overbeek (DLVO) primary energy minima. Interestingly, a large fraction of the deposited heteroaggregates was released by reducing IS because of a monotonic decrease of interaction energy with separation distance for the heteroaggregates at low ISs (resulting in repulsive forces), in contrast to the irreversible deposition of nZVIs. Therefore, the fabricated heteroaggregates could also have high mobility in subsurfaces with saline pore water through continuous capture and release using multiple injections of water with low ISs. Our study was the first to examine the transport of heteroaggregates of a plate-like particle with spherical nanoparticles in porous media; the results have important implications in the use of nanoscale zerovalent iron for in situ soil and groundwater remediation.

## 1. Introduction

Nanoscale zerovalent iron (nZVI) has been one of the most frequently investigated nanomaterials for soil and groundwater remediation during the past two decades [1]. Wang and Zhang [2] conducted a pioneering work and documented that nZVIs can completely dechlorinate several chlorinated aliphatic compounds and a mixture of polychlorinated biphenyls (PCBs) in water. They further postulated that nZVI may be used for in situ remediation of contaminated soil and groundwater through direct injection of nZVIs into a contaminated subsurface [3]. However, the application of nZVIs for in situ soil and groundwater remediation remains challenging, because they readily aggregate and have very low mobility in the aquatic environments [4]. For example, Schrick et al. [5] investigated the transport of nZVIs in soil columns and reported that nZVIs were rapidly aggregated in pore water and efficiently filtered by all the tested soils.

The aggregation of nZVIs is thermodynamically favorable due to attractive interparticle forces including van der Waals (vdW) and magnetic dipolar interactions. One common technique for inhibiting the aggregation of nZVIs and increasing mobility is the adsorption of stabilizer molecules (e.g., surfactants) on nZVI surfaces to increase double layer (DL) repulsion and electrosteric repulsion between them [1,6,7,8]. For example, Xu and Zhao [9] conducted column experiments to examine the transport of nZVIs stabilized by sodium carboxymethyl cellulose (CMC) in a sandy loam soil. They found that the CMC-stabilized nZVIs were highly deliverable in soil columns. Notably, the stabilizer molecules increased the mobility of nZVIs in the porous media not only by increasing dispersion (i.e., inhibiting aggregation) but also by reducing the deposition of nZVIs on aquifer grain surfaces via increasing the electrostatic and electrosteric repulsions between the particles and the collectors [10].

Despite the increase of dispersion and mobility, the stabilizer may reduce the reactivity of nZVIs due to the mask of the nZVI surfaces. Moreover, some stabilizer molecules are toxic and could lead to secondary contamination when they are released into subsurface environments. Alternatively, a variety of templates have been adopted to support nZVIs to inhibit their aggregation without the compensation of masking nZVI surfaces [11]. For instance, Gu et al. [12] used smectite clay layers as templates to synthesize very small isolated regions of nZVIs on clay surfaces, which showed superior reactivity and efficiency for the treatment of nitrobenzene in water. Zhou et al. [13] used chitosan as a dispersing and soldering reagent to distribute fine ZVIs onto bamboo biochar surfaces and the resulted ZVI-biochar composites exhibited enhanced ability for the removal of heavy metals, phosphate, and methylene blue. Whereas the efficiency of the composites of nZVIs with templates has been well documented, the transport of these composites in porous media has received very little attention to date. Moreover, no study has examined the detachment of the colloidal heteroaggregates under transient in solution chemistry to date. Understanding the transport of the composites is critical for the application of the nZVIs for in situ soil and groundwater remediation.

This study systematically examined the transport of novel heteroaggregates of montmorillonite (Mt) microparticles with nZVIs in saturated sand porous media through conducting column experiments. Spherical nZVI particles were generated via the liquid phase reduction method and then attached on the flat Mt surfaces in monolayer. We found that the fabricated Mt-nZVI heteroaggregates significantly reduced the deposition of nZVI on collector surfaces and increased the mobility of nZVIs in the porous media. In particular, the decoration of nZVIs on Mt particle surfaces can cause the monotonic decrease of Derjaguin–Landau–Verwey–Overbeek (DLVO) interaction energy with separation distance at low solution ionic strengths (ISs). Consequently, Mt-nZVI heteroaggregates cannot attach to sand collectors, or the attached heteroaggregates will be released at low ISs because they experience repulsive forces at all separation distances from the collectors. Due to the high efficiency of the Mt-nZVI heteroaggregates in the removal of contaminants such as Cr(VI) from water [14], the fabricated Mt-nZVIs heteroaggregates with such unique micro/nano structure showed great promise for application for in situ soil and groundwater remediation because of their great mobility and meanwhile high efficiency for contaminant removal. 

## 2. Materials and Methods

### 2.1. Synthesis of nZVIs and Heteroaggregates of Mt-nZVIs

The nZVIs were synthesized using the well-known liquid phase reduction method and the heteroaggregates of the Mt-nZVIs were fabricated using the method in Yin et al. [14]. Briefly, a ferrous solution was obtained by adding 21.36 g of FeCl_2_‧4H_2_O into an ethanol–water solution containing 96 mL anhydrous ethanol and 24 mL deionized (DI) water. An NaBH_4_ solution, obtained by adding 12.2 g NaBH_4_ powder into 400 mL DI water, was mixed with the prepared ferrous solution using a pump with stirring. The ferrous solution immediately became black due to generating nZVIs via a reduction of Fe(II) to Fe(0). The resulting nZVI suspension was shaken for 2 h at a speed of 180 r min^−1^ and the nZVI particles were separated from the suspension through vacuum filtration using a 0.22 μm filter. Pure nZVI particles were obtained by washing via 99% ethanol and dried at 85 °C for 10 h. Note that the nZVI particles may be oxidized and shells of iron oxide may be formed, as characterized by using X-ray diffraction and X-ray photoelectron spectroscopy in Yin et al. [14].

Mt powder (Zhejiang Sanding Technology Co., Shaoxing, China) with sizes between 1 and 2 μm was employed to prepare Mt particle suspensions. The nZVI suspension was obtained by dispersing the aforementioned nZVI particles in DI water. Both Mt and nZVI suspensions were sonicated for 15 min to ensure monodispersity and then mixed by carefully transferring the Mt suspension into the nZVI suspension with stirring. The suspension of the Mt-nZVI heteroaggregates was obtained through shaking the suspension of Mt and nZVI particles for 12 h at a speed of 200 r min^−1^. The Mt-nZVI heteroaggregates with different concentrations of nZVI loading on the Mt surfaces can be obtained through changing the concentration ratios of nZVI and Mt suspensions. Three fractions of total nZVI mass per gram of Mt (denoted as FZMs) (i.e., 0.66, 0.33, and 0.165 g g^−1^) were examined. Note that the theoretical maximum loading of monolayer nZVI particles (20 nm) on Mt surfaces (assuming 1.5 μm × 1.5 μm × 500 nm) was reached at FZM = 0.66 g g^−1^. An NaCl solution was added to adjust IS of the suspensions of Mt, nZVI, and Mt-nZVI heteroaggregates, with four solution ISs (i.e., 0.001, 0.01, 0.1, and 0.2 M) investigated.

The morphology and structure of the prepared Mt, nZVI, and Mt-nZVI particles were characterized using scanning electron microscope (SEM, Hitachi S-4800, Tokyo, Japan). The elements of the Mt, nZVI, and Mt-nZVI particles were identified by Energy Dispersive X-Ray Analyzer (EDX, Hitachi S-4800, Tokyo, Japan). The zeta potentials of nZVI, Mt, and Mt-nZVI suspensions at different ISs were determined by measuring electrophoretic mobilities using a Zetasizer Nano ZS (Malvern Instruments Ltd., Southborough, MA, USA). The electrophoretic mobilities were then converted to zeta potential via the Smoluchowski equation. Sizes of these colloids were measured by dynamic light scattering using the Zetasizer Nano ZS. 

### 2.2. Porous Media

Quartz sand (Sigma-Aldrich, St. Louis, MO, USA) with sizes ranging from 300 to 355 μm was adopted to pack the columns. Metal oxides and other impurities were extensively removed from the sand before packing the columns using the method of Elimelech and O’Melia [15]. Briefly, the sand was soaked with 1 M HNO_3_ solution for 12 h, and then rinsed with DI water and dried in an oven at 105 °C. The zeta potentials of sand were measured following the method in previous studies [16,17,18]. Briefly, 7 g cleaned sand was sonicated for 20 min in 12 mL NaCl solution with the ISs of 0.001, 0.01, 0.1, or 0.2 M. Samples of the supernatant were diluted 10-fold in a background NaCl solution and zeta potentials were measured using Zetasizer Nano ZS (Malvern Instruments Ltd., Southborough, MA, USA). 

### 2.3. Transport Experiments

Acrylic columns with a diameter of 1.5 cm and length of 10 cm were employed for carrying out the transport experiments. Cleaned sand was incrementally wet packed in the columns and gently vibrated to avoid layering and air entrapment. Packed porosities (*φ*) were determined to be ~0.4 using $\varphi =1-\frac{m}{\left(\rho V\right)}$, where *m* is packed sand mass, *ρ* is the true density of sand (2.65 g/cm^3^), and *V* is column volume. The packed column was pumped by at least 20 pore volumes (PVs) of the background NaCl solution at a constant approach velocity of 3.5 × 10^−5^ m/s for equilibrium of chemical conditions of the column system. 

Transport experiments were conducted using three phases to examine particle deposition and subsequent release by IS reduction, as adopted in previous studies [17,19,20,21]. In Phase A, 5 PVs of nZVI, Mt, or Mt-nZVI suspension were injected into a saturated sand column for the deposition of these particles in the sand at a given IS. The particle suspension reservoir was agitated and sonicated to minimize aggregation and sedimentation during injection. In Phase B, a particle-free electrolyte solution was injected into the column at the same flow velocity and IS as those in Phase A to displace the unattached particles in pore water. In Phase C, DI water was pumped to examine the release of particles by IS reduction. Concentrations of the nZVI and Mt-nZVI (or Mt) suspensions were determined using a UV-vis spectrophotometer (DU Series 800, Beckman Instruments, Inc., Fullerton, CA, USA) at wavelengths of 240 nm and 508 nm, respectively [22,23].

The deposition of the Mt, nZVI, or Mt-nZVI heteroaggregates was quantified by calculating attachment efficiency *α* via the following expression

(1) $$\alpha =-\frac{2}{3}\frac{d_{c}}{\left(1-\eta_{0}\right)L}\ln\left(\frac{C}{C_{0}}\right)$$

where *d*_c_ is sand diameter, *C* and *C*_0_ are effluent and influent particle concentrations, respectively, *L* is column length, *η*_0_ is single collector contact efficiency determined using the expression in Ma et al. [24]. The values of *C*/*C*_0_ were obtained from breakthrough curves (BTCs) of column experiments through averaging the measurements between PVs of 1.8 and 2 [16,17,25]. 

### 2.4. Calculation of DLVO Interaction Energies

**Figure 1:**
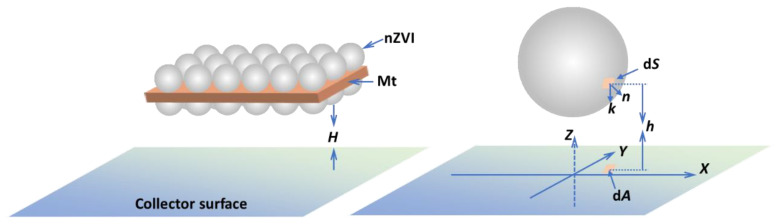
Schematic to illustrate interaction between a modeled Mt-nZVI heteroaggregate or a spherical nZVI particle and collector surface. *H* is separation distance between the heteroaggregate and the surface, d*S* is differential area element on the nZVI particle surface, d*A* is the projected area of d*S* on the planar surface, *h* is local distance between the area element d*S* from the collector surface (i.e., the distance between the d*S* and d*A*), *n* is unit outward normal to the particle surface, and *k* is unit vector normal along the negative z-direction.

The DLVO interaction energies between an nZVI, Mt microparticle or an Mt-nZVI heteroaggregate and sand surface were calculated to interpret the attachment and detachment behaviors of these particles on/from sand surfaces. Similar to Shen et al. [26] and Du et al. [27], nZVI and Mt particles were assumed to be spherical and cuboid shaped, respectively (Figure 1). The Mt-nZVI heteroaggregate was simulated as a cuboid decorated with nanosized spheres on the top and bottom surfaces in monolayer. The sand collector surface was assumed to be planar. The surface element integration (SEI) technique developed by Bhattacharjee and Elimelech [28] was employed to calculate interaction energies between the three types of particles and sand surface. 

Note that we only considered that the top and bottom surfaces of the cuboid were parallel to the planar surface, because only at this interaction configuration can the cuboid particle be stably attached on the surface in the presence of hydrodynamic drag [26]. The DLVO interaction energy (*U*) between the cuboid-shaped particle and the planar surface can be calculated using the following expression according to the SEI technique [10]

(2) $$U\left(H\right)=\left(E\left(h\right)-E\left(h+L\right)\right)S_{D}$$

where *E* is the differential interaction energy between an area element on the top or bottom surface of the cuboid particle and the planar surface, *H* is the separation distance between the cuboid particle and the planar surface, *h* is the local distance between the area element and the planar surface, *L* is the height of the cuboid particle, and *S*_D_ is the area of the top or bottom surface of the cuboid particle. Notably, Equation (2) can also be applied to calculate interaction energies for other shapes of particles such as cubes and pillars. The interaction energies are independent of the shapes of the top and bottom surfaces and are only related to the areas of the surfaces and the height of the particle [10]. 

The differential interaction energy between the area element and the planar surface *E* is calculated as a sum of vdW attraction, DL interaction, and Born repulsion (BR) [29]:

(3) $$E\left(h\right)=E^{vdW}\left(h\right)+E^{DL}\left(h\right)+E^{BR}\left(h\right)$$

The following expressions can be used to calculate *E^vdW^*, *E^DL^*, and *E^BR^* [30,31,32], respectively,

(4) $$\;E^{vdW}\left(h\right)=-\frac{A_{H}}{12\pi h^{2}}$$

(5) $$E^{DL}\left(h\right)=\frac{\varepsilon \varepsilon_{0}\kappa }{2}\left[\left(\psi_{p}^{2}+\psi_{c}^{2}\right)\left(1-coth\left(\kappa h\right)\right)+2\psi_{p}\psi_{c}csch\left(\kappa h\right)\right]$$

(6) $$E^{BR}\left(h\right)=\frac{A_{H}H_{0}^{6}}{48\pi h^{8}}$$

where *A*_H_ is Hamaker constant, *ε*_0_ is permittivity of a vacuum, *ε* is the relatively permittivity of water, *ψ_p_* and *ψ_c_* are zeta potentials of the particle and the sand surface, respectively, *κ* is the inverse Debye screening length, *H*_0_ is the minimum separation distance between the particle and the collector surface, which was taken as 0.158 nm [33]. The values of the Hamaker constant were taken as 6.3 × 10^−21^ J and 2.8 × 10^−20^ J for the for the Mt–water–quartz and iron–water–quartz systems, respectively [26,27,34].

Details about calculating DLVO energies for the sphere–planar surface interaction configuration (Figure 1b) using the SEI technique can be referred to previous studies [28,29,35,36]. Briefly, the spherical nZVI particle surface was discretized into small area elements (d*S*). The total interaction energy was determined as a sum of the differential interaction energy between each area element d*S* and the planar surface [29]

$$U\left(H\right)=\int_{S}E\left(h\right)n\cdot kdS$$

(7) $$=\int_{A}\left(E\left(H+R-\sqrt{R^{2}-\left(x^{2}+y^{2}\right)}\right)-E\left(H+R+\sqrt{R^{2}-\left(x^{2}+y^{2}\right)}\right)\right)dA\;$$

where d*A* is the projected area of d*S* on the planar surface, *n* is the unit outward normal to the spherical particle surface, *k* is the unit vector along the negative *z*-direction, *R* is the radius of the spherical particle. 

The DLVO interaction energy between the Mt-nZVI heteroaggregate and the planar collector surface is equal to the sum of energies for all the loaded spherical nZVI particles and the cuboid particle interacting with the planar surface. This is because, based on the principle of SEI, the interaction energy between an aggregate and a flat surface is mathematically equivalent to superposition of interaction energy between each primary particle and the surface [37]. The calculated DLVO interaction energies were expressed using the unit of kT (k is Boltzmann constant and T is absolute temperature, taken as 295 K).

## 3. Results and Discussion

### 3.1. Characteristics of Particles and Collector Grains

**Figure 2:**
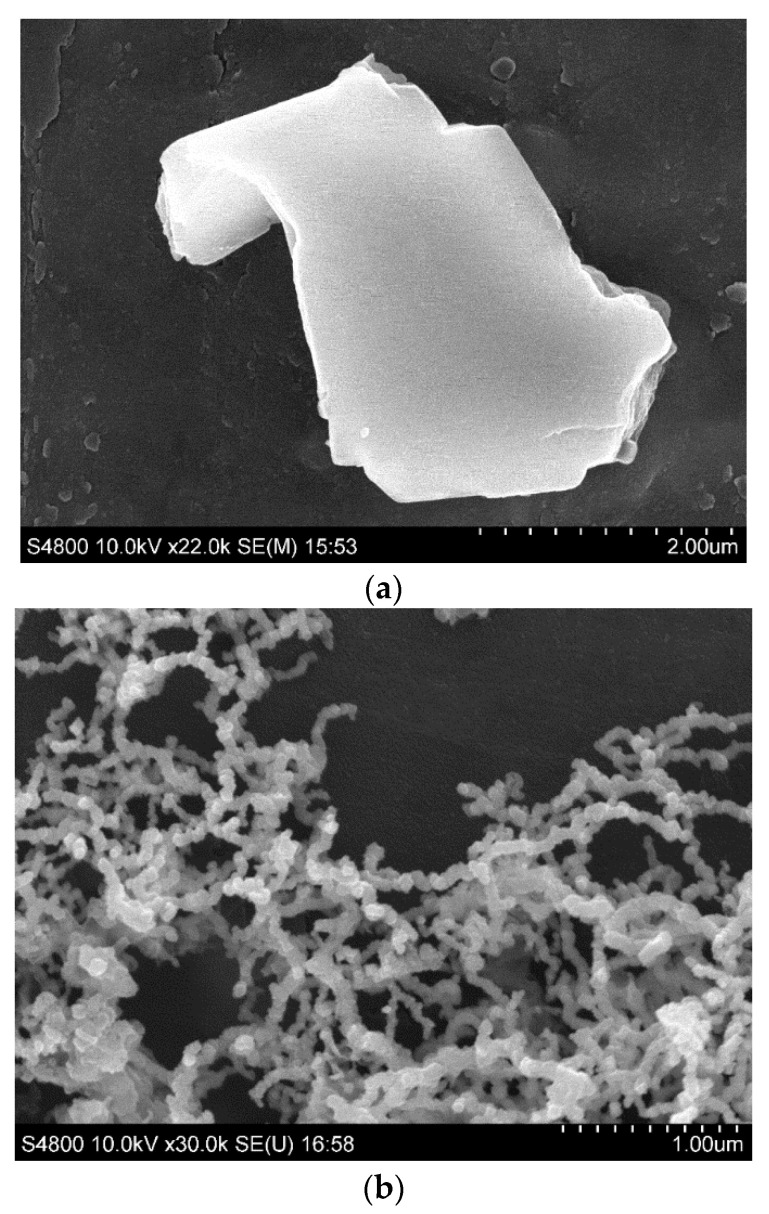

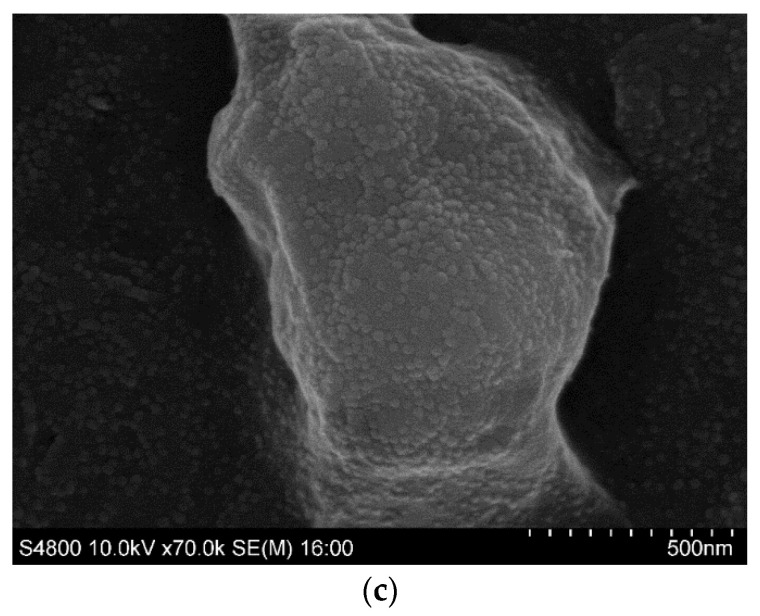
SEM images of (**a**) Mt, (**b**) nZVI particles, and (**c**) Mt-nZVI. The image of Mt-nZVI was taken at FZM = 0.66 g g^−1^.

**Figure 3:**
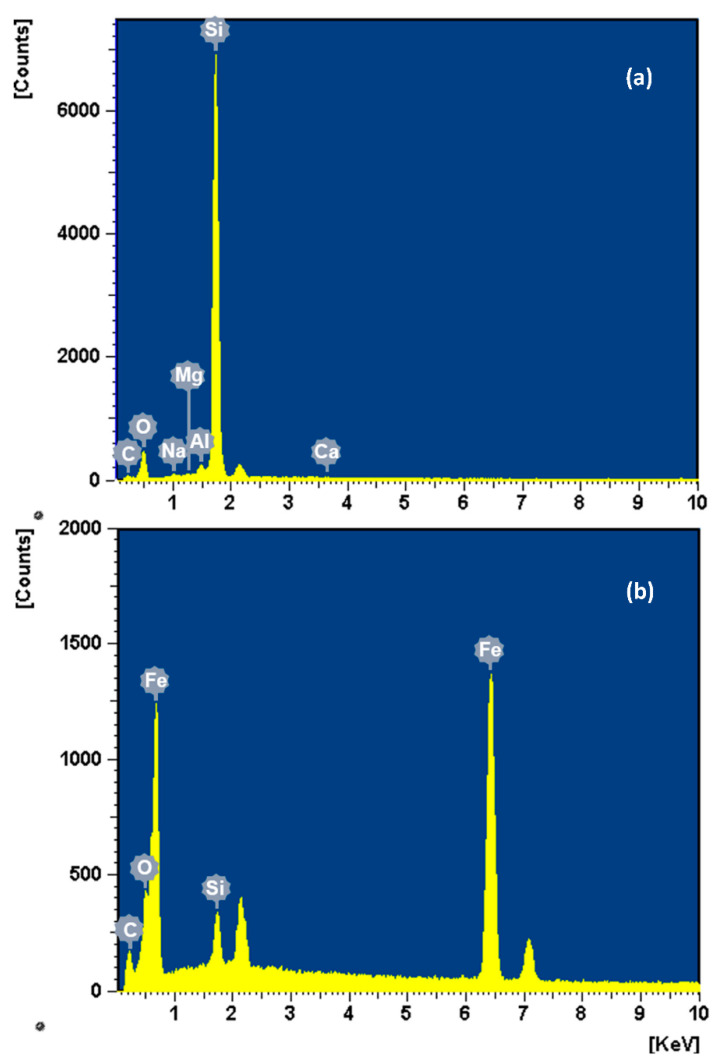
SEM with energy-dispersive X-ray spectroscopy (SEM-EDX) analysis of (**a**) Mt mineral and (**b**) Mt-nZVI heteroaggregate.

Figure 2 presents SEM images of Mt, nZVI, and Mt-nZVI heteroaggregates. The Mt mineral is a 2:1 clay containing two tetrahedral sheets of silica sandwiching a central octahedral sheet of alumina, resulting in plate-shaped Mt microparticles with relatively smooth surfaces. The nZVI particles were aggregated due to attractive vdW and magnetic forces, forming chain-like clusters. The average size of the primary nZVI particles was determined to be 20 nm according to the measurement of at least 100 particles from the SEM images via ImageJ. The nZVI particles were densely and evenly distributed on the Mt template in monolayer at FZM = 0.66 g g^−1^. The EDX analysis further confirmed successful loading of nZVIs on Mt surfaces, as indicated by the existence of considerable Fe elements (Figure 3). Table S1 of the Supplementary Material shows that the zeta potentials of the Mt microparticles and sand were negative at the four tested ISs, in agreement with observations in previous studies [26,38,39]. Consequently, the deposition of Mt on the sand surfaces was *unfavorable* due to the existence of repulsive DLVO energy barriers. In contrast, the zeta potentials of nZVIs were positive, indicating that positive charges existed on nZVI surfaces and oxidation occurred. Therefore, the deposition of nZVI particles on Mt and sand surfaces were *favorable* due to both attractive vdW and DL interactions. The zeta potentials of the Mt, sand, and nZVIs were less negative or positive at a higher IS due to charge screening effects (from −45.5 mV, −31.1 mV, 19 mV at 0.001 M to −22 mV, −6.7 mV, and 2.7 mV at 0.2 M, respectively) [14]. This is because according to the Gouy–Chapman model, higher IS results in a thinner double layer and accordingly a smaller zeta potential. The zeta potentials were less negative for the Mt-nZVI particles with a higher loading of nZVIs, because loading of the positively charged nZVIs reduced the total negative charges on Mt-nZVI surfaces [14]. 

Table S1 of the Supplementary Material shows that the sizes of the Mt and Mt-nZVI heteroaggregates were larger at a higher IS (particularly at the highest tested IS values, i.e., 0.2 M), indicating an aggregation of the Mt and Mt-nZVI particles. It is interesting to note that the sizes of the Mt-nZVIs were evidently larger compared with those of the Mt at a given IS. This is because the loading of positively charged nZVIs on the negatively charged Mt surfaces reduced the repulsive energies between Mt particles, resulting in aggregates of Mt-nZVIs with larger sizes. Due to a similar reason, the sizes of the Mt-nZVIs increased with increasing loading of nZVIs on Mt surfaces at a given IS.

### 3.2. Particle Deposition in Sand Porous Media

**Figure 4:**
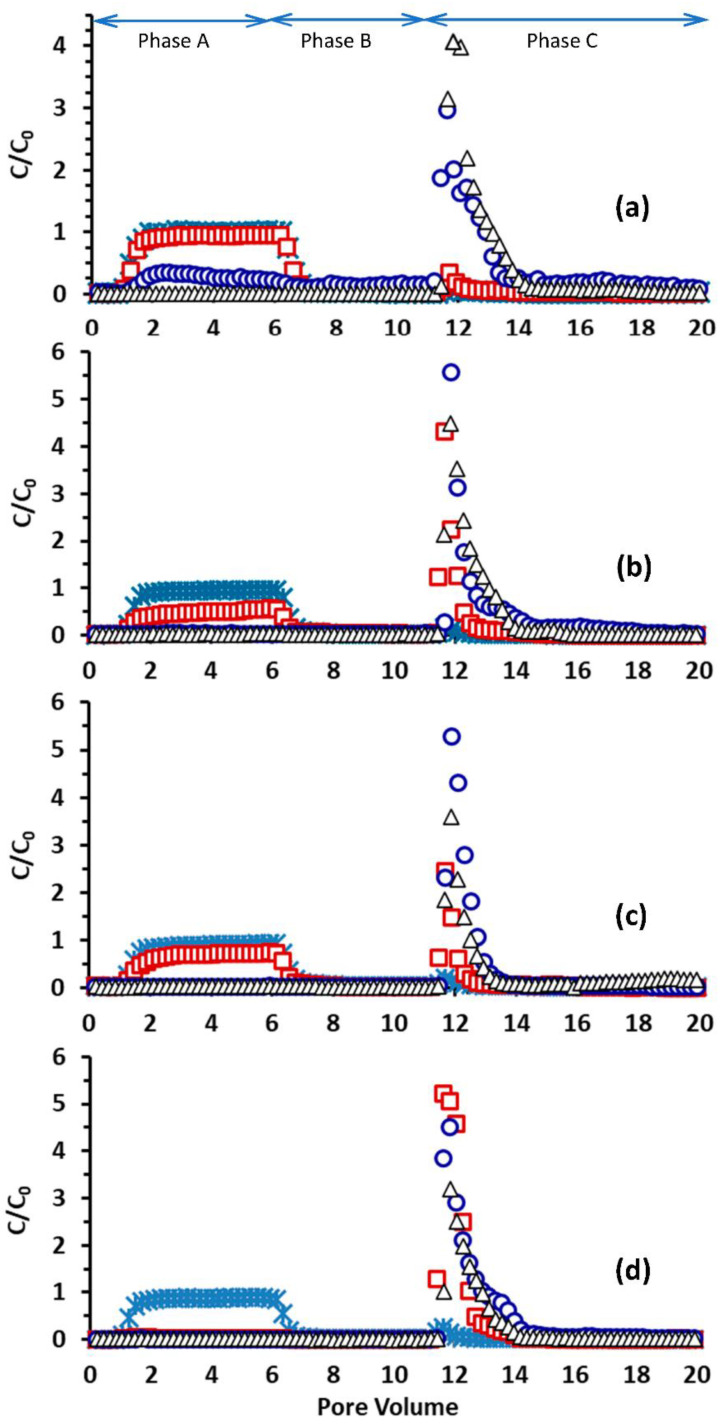
BTCs for transport of (**a**) Mt and (**b**–**d**) Mt-nZVI heteroaggregates in sand columns at different ISs (asterisk, 0.001 M; square, 0.01 M; circle, 0.1 M; triangle, 0.2 M). The FZMs were 0.165, 0.33, and 0.66 g g^−1^ in (**b**–**d**), respectively.

Figure 4 presents BTCs of Mt microparticles and Mt-nZVI heteroaggregates from sand column experiments at different ISs. A complete deposition of nZVIs (i.e., no nZVI particles in the effluents) was observed at all solution ISs (data not shown), implying a very low mobility of nZVI particles in porous media. This observation has been frequently reported in previous studies [40,41,42,43,44]. As shown in Figure 2, the nZVI particles were aggregated and chain-like aggregates were formed because of attractive vdW and magnetic forces. Large nZVI aggregates were readily retained in the sand porous media via straining [45,46,47,48,49]. Bradford et al. [45,46] illustrated that strained colloids were mainly located at the column entrance, causing hyper-exponential retention profiles. Indeed, we found that the color of the packed beds at the column inlets changed from white to black due to the enrichment of nZVIs via straining (data not shown). In addition to straining, nZVI particles were oxidized (as indicated by the positive charges mentioned previously), and thus were also favorable for deposition on sand surfaces due to an attractive DL interaction, causing extremely low mobility in the packed beds. 

Figure 4 shows that the retention of Mt increased with increasing IS in Phase A; particularly, complete deposition occurred at the highest IS tested in this work (i.e., 0.2 M). The DLVO interaction energies between an Mt cuboid (assuming 1.5 μm × 1.5 μm × 0.5 μm) and the sand surface were calculated using the aforementioned SEI technique and shown in Figure S1 of the Supplementary Material. The maximum energy barriers *U*_max_ were calculated to be 73,242, 163,308, 95,907, and 0 kT at 0.001, 0.01, 0.1, and 0.2 M, respectively. The calculated secondary minimum depths *U*_sec_ were 4.56, 75.08, 1386.6, and 0 kT at 0.001, 0.01, 0.1, and 0.2 M, respectively. These calculations showed that the Mt microparticles were favorably attached at the primary minima at 0.2 M due to the absence of a repulsive energy barrier, resulting in complete deposition. In contrast, the maximum energy barriers were orders of magnitude larger than the average kinetic energy of a colloid in solution (i.e., 1.5 kT) at IS ≤ 0.1 M. Hence, colloids should be retained via the secondary minimum association at these ISs. 

It should be noted that particles that are attached at the secondary minima will be detached when DI water is used to reduce solution IS due to reduction and elimination of the secondary minima from interaction energy profiles [20,29,50]. However, as will be shown later in the paper, only a small fraction of deposited colloids at IS ≤ 0.1 M were reversible when reducing solution IS in Phase C (e.g., only 6% at 0.001 M). One possible reason is that the theoretical calculations did not consider the surface chemical heterogeneities. The EDX analysis showed that Mg and Ca elements existed in sand (Figure 3). The Mt particles could be irreversibly attached on the sand surfaces via a cation bridge due to these bivalent cations even at the lowest IS [51]. In addition, atomic force microscopy (AFM) examinations in Figure S2 of the Supplementary Material showed that the quartz sand surfaces were very rough, similar to the observations in previous studies [10,17,52]. The Mt microparticles could be irreversibly attached at concave locations of sand surfaces via primary minimum association [52]. The irreversible attachment at concave surfaces is because the primary minima are deep at all solution ISs at the concave surfaces. Although nanoscale protruding asperities (NPAs) also increase particle deposition by reducing repulsive energy barrier, the deposited particles atop NPAs are reversible to the reduction of solution IS because the interaction energy decreases monotonically with separation distance at these sites.

It is worthwhile mentioning that the calculated interaction energies for the Mt cuboid (Figure S1 of the Supplementary Material) were orders of magnitude larger than those for particles with other shapes (e.g., sphere, spheroid) reported in the literature [26,53,54,55,56]. This can be explained using the concept of “interaction volume” proposed by Huang et al. [57]. The interaction volume is defined as the volume between the leading surface of a particle and the interacting substrate surface. The interaction energy increases with decreasing interaction volume. The interaction volume reaches a minimum for the cuboid particle with a flat bottom surface approaching a planar collector surface compared with particles with other shapes such as sphere and spheroid. Therefore, the interaction energies are significantly larger for the Mt cuboids.

**Figure 5:**
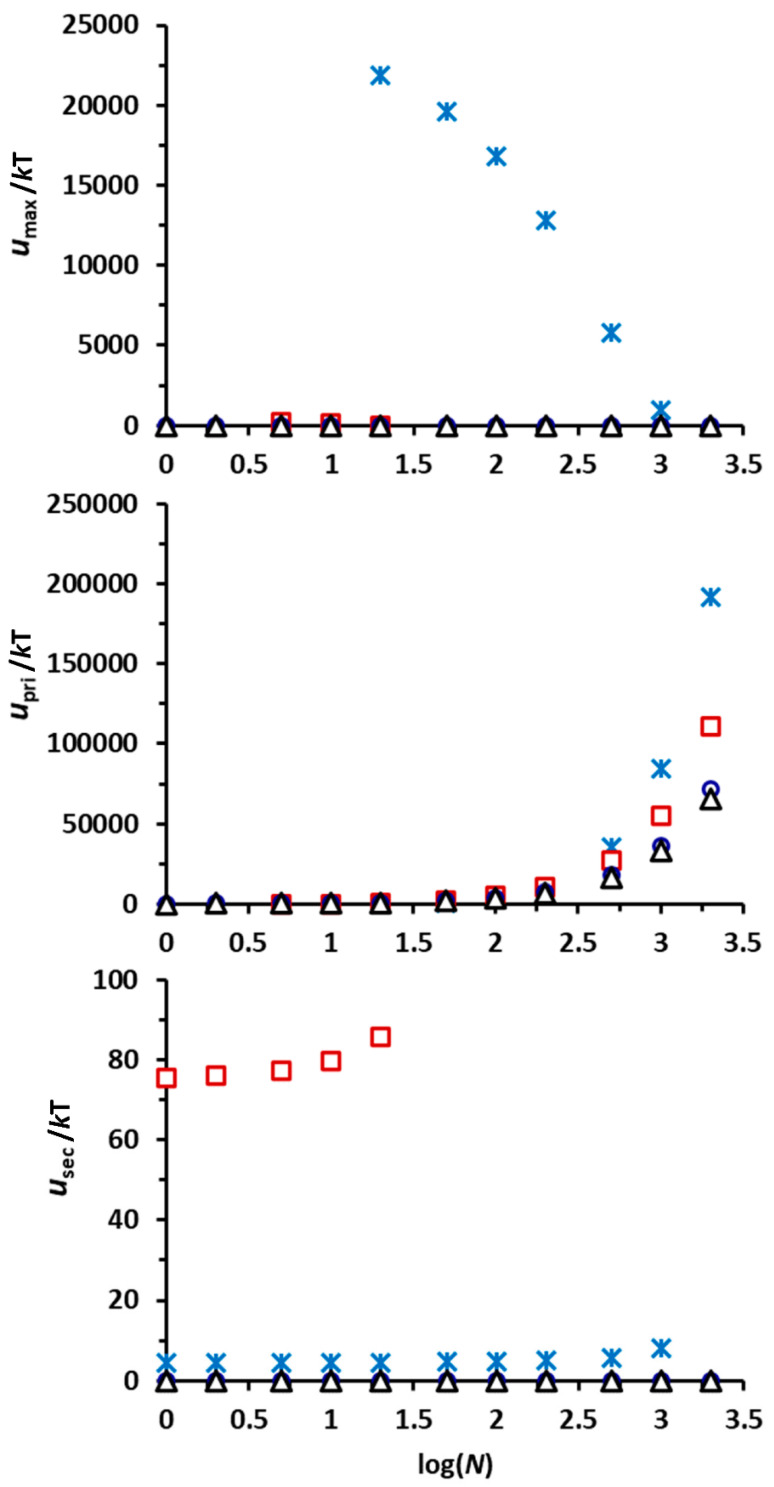
Calculated maximum energy barrier (*U*_max_), primary minimum depth (*U*_pri_), and secondary minimum depth (*U*_sec_) for interactions between a heteroaggregate of a cuboid Mt loaded with different numbers of spherical nZVIs (*N*) and a planar surface at different ISs (asterisk, 0.001 M; square, 0.01 M; circle, 0.1 M; triangle, 0.2 M). The length or width of the cuboid is 1.5 μm and the height is 500 nm. The diameter of the spherical nZVIs is 20 nm.

Figure 4 shows that the loading of nZVIs on the Mt surfaces increased the deposition of Mt-nZVI heteroaggregates in sand compared with Mt. The deposition of Mt-nZVI heteroaggregates increased with increasing the FZM. For example, while complete deposition occurred only at IS = 0.2 M for the Mt particles (i.e., FZM = 0), complete deposition existed at IS ≥ 0.1 M for FZM = 0.165 g g^−1^ and FZM = 0.33 g g^−1^, and at IS ≥ 0.01 M for FZM = 0.66 g g^−1^. The DLVO interaction energy calculations in Figure 5 showed that the calculated values of *U*_max_ decreased while *U*_sec_ increased with an increasing number of nZVI loadings on the Mt surface (*N*) at IS ≤ 0.01 M. Accordingly, both primary and secondary minimum attachments of Mt-nZVI particles increased with increasing the value of *N* at IS ≤ 0.01 M. The repulsive energy barriers disappeared, and only primary minima existed in the interaction energy profiles at IS ≥ 0.1 M for *N* ≥ 1. The primary minimum depth increased with the increasing value of *N*. Hence, the Mt-nZVI heteroaggregates experienced larger adhesive forces with a higher loading concentration of nZVIs. This explains that the primary minimum attachment of Mt-nZVI particles increased with an increasing value of *N* at IS ≥ 0.1 M. The primary minimum attachment could be further increased if roughness and charge heterogeneity on sand surfaces are included, as has been mentioned previously. 

Figure S3 of the Supplementary Material presents the calculated values of attachment efficiency α using Equation (1) for Mt and Mt-nZVI heteroaggregates with different nZVI loadings at different solution ISs. The calculated values of α increased with increasing FZM and solution IS. Particularly, the values of α reached unity (i.e., complete deposition) at IS = 0.2 M for all examined FZM values, at IS = 0.1 M for FZM ≥ 0.165 g g^−1^, and 0.01 M for FZM = 0.66 g g^−1^. The result indicates that all Mt and Mt-nZVI particles that collided with the sand collectors were attached on the collector surfaces in these cases. The values of α were < 0.15 at IS = 0.001 M for all values of FZM, at IS = 0.01 M for FZM ≤ 0.33 g g^−1^, and at IS = 0.1 M for FZM = 0 (i.e., Mt). These results clearly demonstrate that the fabricated micro/nano structure significantly increased the mobility of nZVI particles at the typical IS range of groundwater (i.e., IS ≤ 0.01 M) [58]. This is because the attractions between nZVI particles and collectors were reduced or even eliminated by the repulsion between the Mt and the grain surfaces. 

### 3.3. Particle Release in Sand Porous Media

Figure 4 shows that a fraction of the Mt and Mt-nZVIs deposited in sand in Phase A were released in Phase C upon IS reduction. As mentioned previously, colloids that are attached at the secondary minima will be released by IS reduction because the secondary energy minimum decreases with increasing IS [19,20,50,59,60]. In addition, colloids that are attached atop NPAs via primary minimum association can be detached by decreasing solution IS [10,22]. This is because the NPAs can significantly reduce the depth of primary minima and cause a decrease of primary minimum depth with decreasing IS. Considerable NPAs existed on the sand surface due to the fractal of sand surface roughness (Figure S2 of the Supplementary Material), which were thus responsible for the reversible attachment of the Mt particles and the Mt-nZVI heteroaggregates. The reversible attachment can be further increased when the Mt surfaces also exist NPAs. Notably, no detachment occurred in Phase C, when only nZVI particles were injected into the columns (data not shown), indicating the irreversible deposition of nZVI particles.

**Figure 6:**
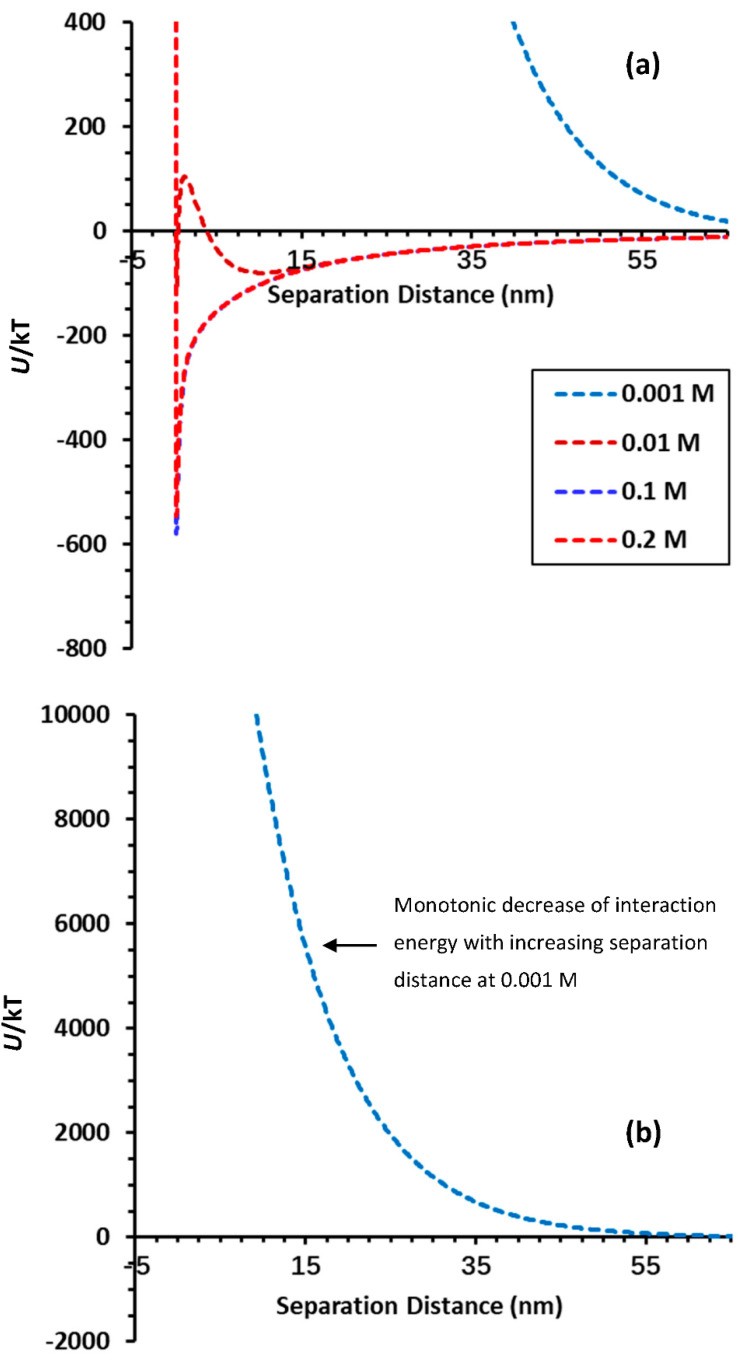
Calculated DLVO interaction energy profiles for a cuboid Mt (1.5 μm × 1.5 μm × 500 nm) loading with 20 nZVI particles interacting with a planar sand surface at different solution ISs; (**b**) is re-plotted profiles of (**a**) to highlight the monotonic decrease of interaction energy with increasing separation distance at 0.001 M.

It is worthwhile mentioning that the loading of nZVIs on Mt surfaces can cause the detachment of Mt-nZVI aggregates even from planar surfaces (i.e., without the presence of nanoscale heterogeneities). For example, Figure 6 presents calculated DLVO interaction energy profiles for an Mt cuboid (1.5 μm × 1.5 μm × 500 nm) decorated with 20 nZVI particles interacting with a planar sand surface at different solution ISs. Only primary minima exist at energy profiles for solutions with IS = 0.1 and 0.2 M, illustrating that Mt-nZVI heteroaggregates were favorably attached at primary minima. The interaction energy decreases monotonically with an increasing separation distance at 0.001 M, indicating that colloids experience repulsive forces at all separation distances. Consequently, the Mt-nZVIs initially attached at IS ≥ 0.1 M via a primary minimum association will be detached by decreasing solution IS. The monotonic decrease of interaction energy with an increasing separation distance is because the primary minimum attraction between the nZVIs and sand surface is eliminated by the repulsion between the Mt and the sand surface. This reflects the fact that nZVIs played a role similar to NPAs in the manipulation of the interaction energies between Mt microparticles and sand collectors. Although colloids attached on nanoscale surface charge heterogeneity via primary minimum association have been traditionally regarded as chemically reversible, Shen et al. [35] illustrated that nanoscale charge heterogeneity cannot solely cause the detachment upon IS reduction, because the charge heterogeneity actually increases the primary minimum depth and the adhesive force. However, the presence of charge heterogeneity on NPAs can increase the attachment on the NPAs and the subsequent detachment during transient in solution chemistry.

**Figure 7:**
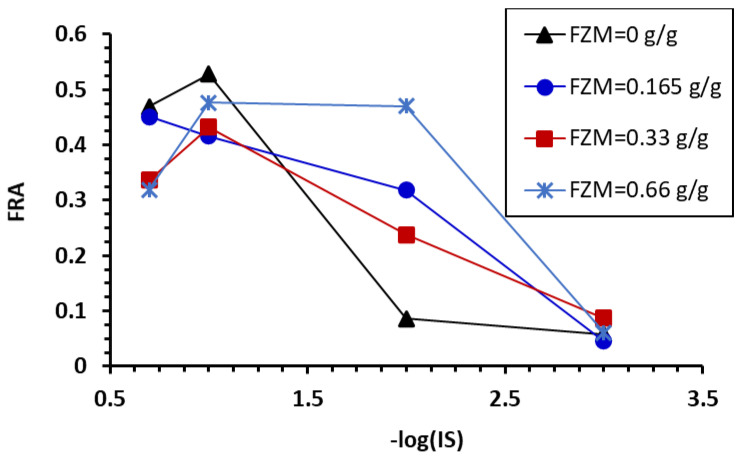
Calculated fraction of reversibly attached Mt and Mt-nZVI (FRA) as a function of solution IS at which the particles were attached in Phase A. FRA = *M*_C_/(1−*M*_AB_), where *M*_AB_ and *M*_C_ are the fraction of colloids recovered from Phase A and B and Phase C, respectively.

Figure 7 presents the calculated fractions of Mt or Mt-nZVIs deposited at different ISs in Phase A that were detached in Phase C (i.e., reversibly attached colloids, denoted as FRA) due to IS reduction. Less than 10% of the attached Mt particles and Mt-nZVI heteroaggregates were released when they were initially attached at IS = 0.001 M. This indicated that the main retention mechanism of Mt and Mt-nZVIs were irreversible attachment at the primary minima (e.g., retention at concave locations and chemical heterogeneities) instead of attachment at the secondary minima or NPAs (Li et al., 2017). In contrast, more than 30% of the Mt-nZVI heteroaggregates retained at IS = 0.1 and 0.2 M were released upon the reduction of solution IS, although the reversibility of the Mt-nZVI heteroaggregates was slightly decreased compared with the Mt particles. Therefore, despite complete retention occurring for the Mt-nZVI heteroaggregates at IS ≥ 0.1 M, a large fraction of attached colloid can be re-entrained into the pore solution during transient in solution chemistry. Recent microscopic observations [61,62,63,64] showed that such continuous capture and release could cause the very long-distance travel of colloids in porous media. 

### 3.4. Implications

Our work has important implications for the delivery of nZVI suspensions for the in situ remediation of soil and groundwater contamination. We showed that a fabricated micro/nano structure (i.e., a plate-shaped microparticle decorated with nanosized spherical nZVIs in monolayer) can significantly enhance nZVI mobility in subsurface environments because the attractions between nZVIs and collector grains are reduced or even eliminated by repulsive forces between the plate-shaped microparticle and the grain surfaces. The mobility of heteroaggregates may be further enhanced using hollow microparticles as carriers to decrease density and sedimentation. Such micro/nano heteroaggregates may be feasible for remediation in subsurfaces with pore solutions of low ISs due to their high mobility. These heteroaggregates may also be applied for remediation in subsurfaces with saline pore water through multiple injections of water with low IS to enhance spreading via continuous capture and release. Interestingly, our additional experiments (Figure S4 in the Supplementary Material) showed that the Mt microparticles can even carry the aggregated nZVIs to transport throughout porous media. This suggests that the mobility of nZVIs in subsurfaces can be enhanced by simply adding microparticles with high mobility into the nZVI aggregate suspension to reduce the deposition of the nZVI aggregates on collector grains. As Yin et al. [14] showed a high efficiency of the Mt-nZVI heteroaggregates for the removal of contaminants such as Cr(VI) from water, the fabricated Mt-nZVIs heteroaggregates showed great promise for application for in situ soil and groundwater remediation because of their great abilities to reach contamination sites and their high efficiency for the removal of contaminants. The use of Mt-nZVI heteroaggregates for in situ remediation of soil and groundwater is an ongoing topic, but is beyond the scope of this study.

## 4. Conclusions

Novel heteroaggregates of plate-like Mt microparticles decorated with spherical nZVIs in monolayer were fabricated, and the transport of the Mt-nZVI heteroaggregates and nZVIs in saturated sand porous media at different ISs was investigated by conducting column experiments. Whereas complete deposition occurred for the nZVIs, significant transport through the columns was observed for the Mt-nZVIs at IS = 0.001 M for all tested FZM values and at IS = 0.01 M for FZM ≤ 0.33 g g^−1^. The significant mobility was because the attractions between the nZVIs and the sand collectors were reduced or even eliminated by the repulsions between the Mt microparticles and the collector surfaces, inhibiting the deposition of the Mt-nZVIs. Whereas the complete deposition of Mt-nZVI heteroaggregates occurred at IS ≥ 0.1 M for all values of FZM due to favorable attachment at the primary minima, a large fraction of the deposited heteroaggregates can be released by IS reduction. The release of heteroaggregates with decreasing solution IS is because the interaction energy decreased monotonically with the separation distance for the heteroaggregates at low ISs (i.e., the heteroaggregates experienced repulsive forces) and no interaction energy wells existed in the energy profiles. Therefore, the Mt-nZVIs that were attached at high ISs via a primary minimum association were detached by reducing solution IS. Our study was the first to examine the release of colloidal heteroaggregates during transient in solution chemistry, and the observed continuous capture and release can lead to the significant mobility of the heteroaggregates in subsurface environments during transient in solution chemistry. Therefore, the composites with the unique micro/nano structure show promise for the in situ remediation of contaminated soil and groundwater since they also had high efficiency for the removal of contaminants.

## Data Availability

Data is contained within the article or supplementary material.

## Supplementary Materials

The following supporting information can be downloaded at: https://www.mdpi.com/article/10.3390/toxics10060332/s1, Figure S1: DLVO interaction energy profiles between a Mt cuboid particle and a planar sand surface at different solution ISs. *k* is Boltzmann constant and T is absolute temperature; Figure S2: A typical AFM image of sand surface; Figure S3: Calculated values of attachment efficiency α as a function of FZM at different solution ISs using the BTCs in Figure 4; Figure S4: SEM images of the particles in the effluents for column experiments with injection of mixture of Mt suspension and nZVI aggregate suspension; Table S1: Sizes and zeta potentials of Mt, nZVI, Mt-nZVI, and sand collectors at different ISs.
