# Supplementary material for: Significant Mobility of Novel Heteroaggregates of Montmorillonite Microparticles with Nanoscale Zerovalent Irons in Saturated Porous Media
Source: Toxics. 2022 Jun 17;10(6):332. doi: 10.3390/toxics10060332 (PMC9227587; doi:10.3390/toxics10060332)
Supplement: Supplementary file 1 [file toxics-10-00332-s001.zip › toxics-1694723-supplementary.pdf]

toxics MDPI

Supplementary Materials: Significant Mobility of Novel Heteroaggregates of Montmorillonite Microparticles with Nanoscale Zerovalent Irons in Saturated Porous Media

Chongyang Shen, Jinan Teng, Wenjuan Zheng, Dong Liu and Ke Ma

Table S1. Sizes and zeta potentials of Mt, nZVI, Mt-nZVI, and sand collectors at different ISs. The values of FZM were 0.66 g g^{-1}, 0.33 g g^{-1}, 0.165 g g^{-1}, and 0.0825 g g^{-1} for A, B, C, and D, respectively.

IS (M)	Particle size (nm)						Zeta potential (mV)						
	Mt	nZVI	Mt-nZVI				Sand	Mt	nZVI	Mt-nZVI			
			A	B	C	D				A	B	C	D
0.001	965	1210	1652	1542	1376	1210	−45.5	−31.1	19	−30.3	−33.2	−34.4	−36.8
0.01	1256	3080	2427	2213	2185	1878	−36.5	−29.5	9.8	−28.3	−30.5	−31.5	−34.6
0.1	1689	4350	3003	2654	2497	2054	−26.8	−17.2	4.7	−24.1	−28.2	−28.8	−29.4
0.2	2218	7360	3163	3027	2930	2624	−22	−6.7	2.7	−22.1	−25.3	−25.4	−27.3

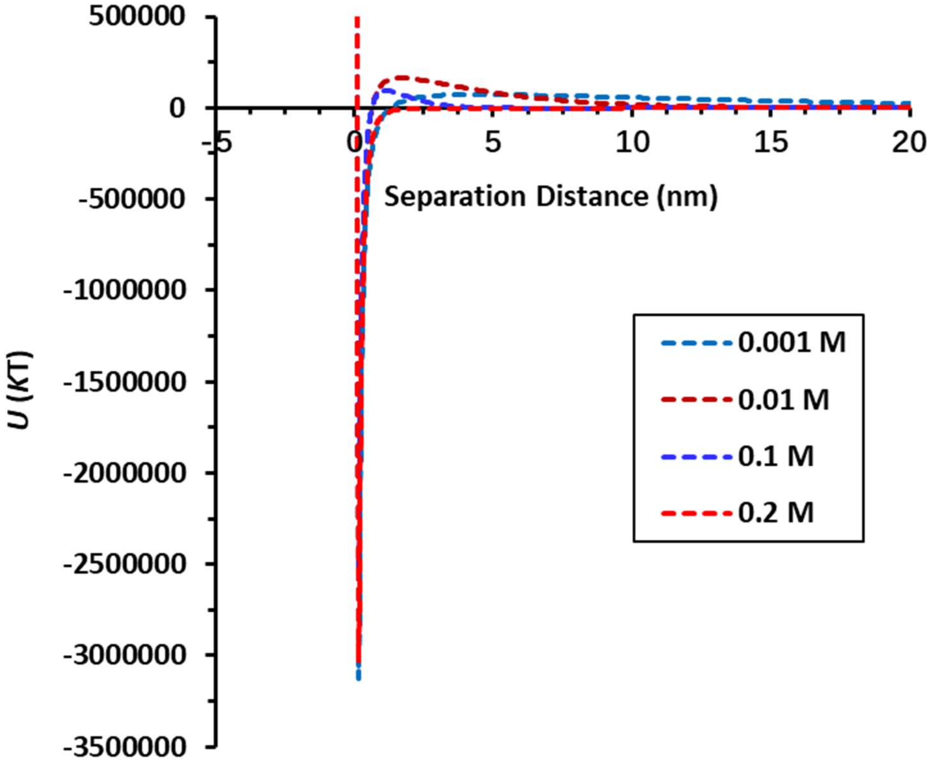

Figure S1. DLVO interaction energy profiles between a Mt cuboid particle and a planar sand surface at different solution ISs. *k* is Boltzmann constant and T is absolute temperature.

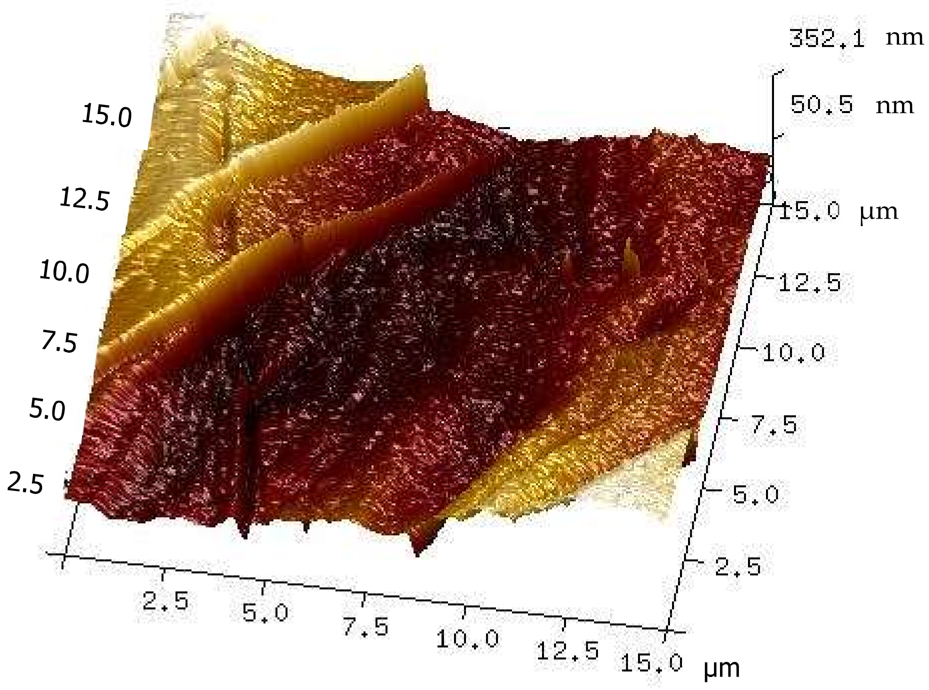

Figure S2. A typical AFM image of sand surface.

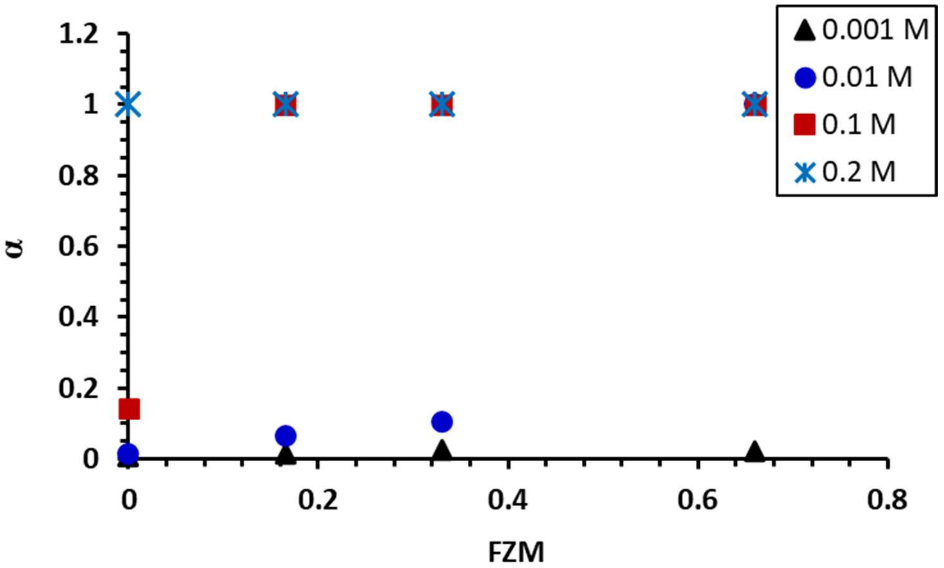

Figure S3. Calculated values of attachment efficiency α as a function of FZM at different solution ISs using the BTCs in Figure 4.

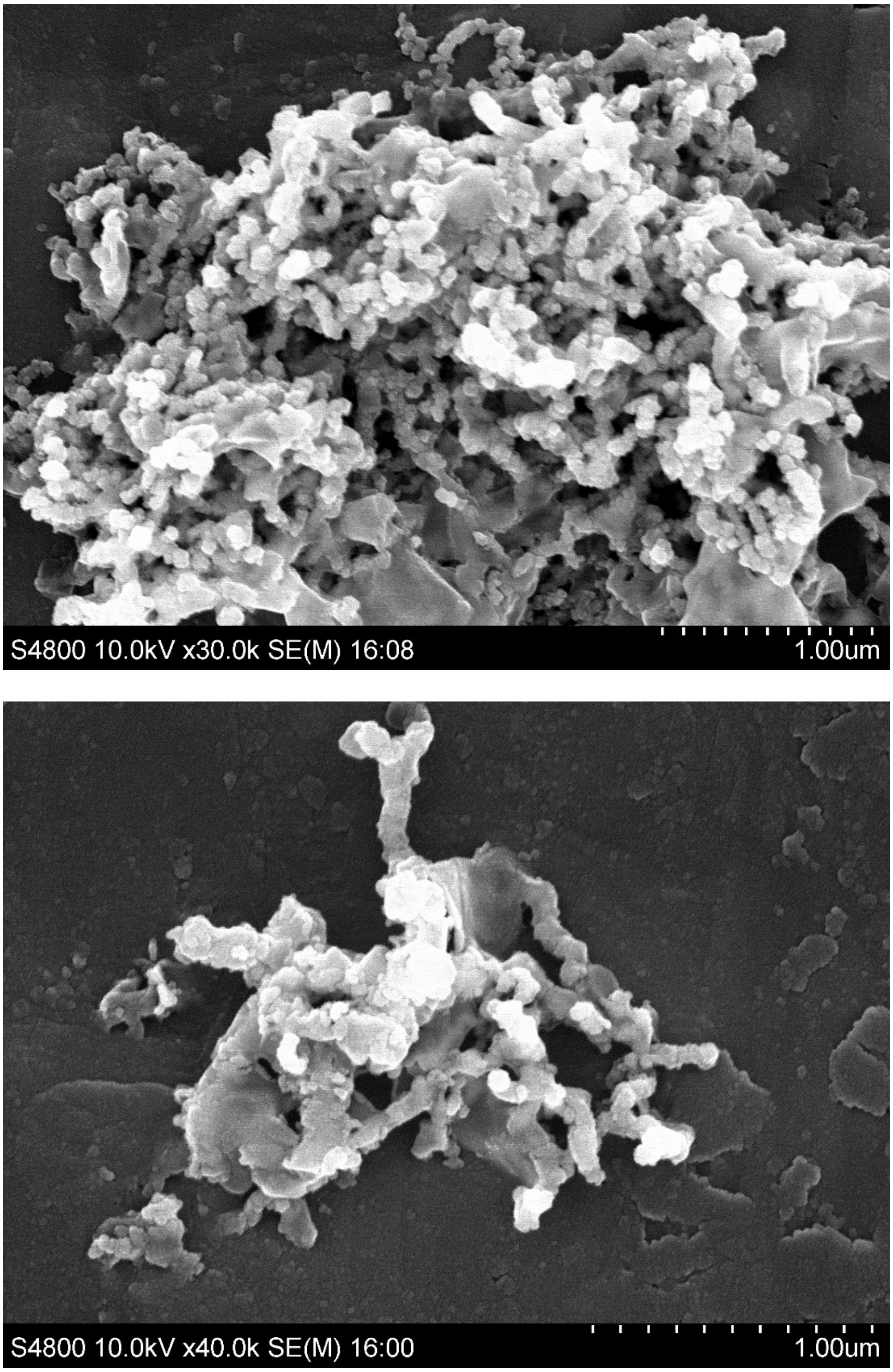

Figure S4. SEM images of the particles in the effluents for column experiments with injection of mixture of Mt suspension and nZVI aggregate suspension.
